# Is pea our hidden allergen? An American pediatric case series

**DOI:** 10.1016/j.jacig.2023.100090

**Published:** 2023-03-16

**Authors:** Racha Abi-Melhem, Yasmin Hassoun

**Affiliations:** a Staten Island University Hospital, Staten Island, NY; b Division of Allergy and Immunology, Department of Pediatrics, Cincinnati Children's Hospital Medical Center, University of Cincinnati College of Medicine, Cincinnati, Ohio

**Keywords:** Green pea, *Pivum sativum,* Dun pea, *Pivum sativum sativum var. arvense,* legumes, allergens, *Pisum sativum* 1, *Pisum sativum* 2, Vicilin, Covicilin

## Abstract

Plant-based diets, consisting of legumes, are becoming increasingly a diet trend, thus a focus for food manufacturers as a source of protein. Allergy to legumes, specifically to green pea and dun pea, have been emerging. There is currently no data on pea allergy in the United States. As such, and with the progressive increase in pea/pea proteins inclusion into foods, we present in this case series children with allergic reactions to foods containing green peas or pea-protein.

High-protein diets have been taking center stage in the world of dietetics and health nutrition as an efficient strategy for weight loss.^1^^,^^2^ Plant-based diets consisting of legumes are gaining popularity as a clean source of protein. Food allergies have also driven the pursuit for dairy and nut-based product alternatives. Pea protein is now becoming an attractive protein-based “allergen-free” product that is taking many forms and is readily available to the public. Pea protein is accessible in various goods such as but not limited to pea-based milk, pea protein powder, and gluten-free pasta alternative, and it is also a main ingredient of vegan “meat.”

Peas belong to the legume family, which includes soybeans, lupins, and peanuts.^3^ There are multiple published reports of legume allergies.^4^, ^5^, ^6^, ^7^, ^8^ There are different subspecies of green pea, with green pea (*Pisum sativum*) and Dun pea (*Pisum sativum sativum var. arvense*) being the most clinically relevant, as green pea is readily available to the consumer and Dun pea is the form of pea protein that has been incorporated into manufactured food.^9^ Dun pea proteins are found in the constituents of minced steak, in the breadcrumbs used to coat meats, in pharmaceutical products, and in specialized food for athletes.^3^ Peanut is the only legume that requires mandatory labeling in the United States, allowing consumers with peanut allergy to easily identify it and consequently avoid any allergic reaction. Pea protein is not included on food labels as a potential food allergen in the European Union, Canada, or the United States,^10^ thus making it a masked potential cause of allergic reactions.

Green pea and Dun pea allergies have been emerging and reported in several studies and case reports.^3^^,^^7^^,^^8^ The 2 main allergens found in peas are *Pisum sativum* 1 and *Pisum sativum* 2, which are members of the *Vicilin* and *Covicilin* families, respectively. A published Canadian case series reported 6 children with allergic reactions to food containing pea ingredients. Many of these reactions were severe and likely met the definition of anaphylaxis according to the consensus criteria. Some of those children had a concurrent peanut or legume allergy.^10^ In addition, 2 children with anaphylaxis in response to Dun pea and others with allergic reactions (asthma exacerbation, urticaria, and laryngeal angioedema) to Dun pea were reported in the French Allergy Vigilance Network from 2002 to 2012.

Currently, there are no data on Dun pea allergy in the United States and very few data regarding green pea allergy. As such, with the progressive increase in inclusion of pea or pea proteins in foods, we report 6 children who presented to a hospital-based allergy clinic with allergic reactions to foods containing green peas or pea protein. Table I lists patient characteristics and serum IgE levels for relevant food allergens. Table II lists serum IgE levels for other food allergens and completed oral food challenge results.

**Table I:** Patient characteristics

Patient no.	Sex	Age (y)	Food listed/ pea ingredient	Reaction reported	Pea-specific IgE level (kU/L)	Peanut allergy
1	M	10	Veggie burger	Hives and vomiting	8.75	Yes
2	F	3	Green peas	Vomiting, sneezing, right periorbital edema and erythema, perioral erythema, hives	7.22	No
3	M	2	Green peas		34.5	Yes
4	M	15	Vegan milk, chicken, nuggets, almond milk		6.1	No
5	M	2	Oat milk mint chip ice cream	Perioral hives, throat clearing, and swelling	31	Yes
6	M	7	Green peas	Facial angioedema and hives	73.4	No

**Table II:** Additional patient characteristics

Patient no.	Comorbidities	Other food allergies	Serum IgE level of other allergens (kU/L)∗	Oral food challenges
1	Asthma	Tree nut	Almond: 4.2	None
	Eczema	Peanut	Brazilian nut: 0.9	
			Cashew: 0.38	
			Pecan: 0.33	
			Walnut: 0.83	
			Pistachio: 1.42	
			Hazelnut: 3.55	
			Peanut: 87.8	
2	Allergic rhinitis	Eggs	Egg white: 10.40	Egg, baked (passed)
			Green bean: 0.64	Green bean (passed)
3		Fish	Cod: 21	None
		Peanuts	Salmon: 33.5	
		Salmon	Tilapia: 24.5	
			Tuna: 3.92	
			Peanut: 6.22	
4		Cashew	—	None
5		Peanuts	Coconut: 0.98	None
			Peanut: 4.82	
			Tapioca: <0.10	
6	Eosinophilic esophagitis	Lentils	Lentil: 30	None
	Allergic rhinitis			

∗ Serum IgE level of allergens tested during the office visit for evaluation and follow-up of food allergies other than pea allergy.

## Case series

### Patient 1

A 10-year-old boy with a known medical history of asthma and eczema presented for follow-up of food allergy to peanuts and tree nuts. The patient reported a new reaction to the Beyond Meat veggie burger brand (Beyond Meat, El Segundo, Calif) since his last office visit. The first ingredient was pea protein. Possible allergens listed on the ingredient list were rice protein, yeast, cocoa butter, and sunflower lecithin, all of which had been previously tolerated by the patient and which he has continued to eat after the reaction. Interestingly the packaging was labeled with a caution that peas are legumes and those with a peanut allergy must be careful about ingesting them. The product label stated that it did not contain any peanuts or tree nuts. The patient broke out in hives that were associated with multiple episodes of vomiting. He was taken to the emergency department, where he was treated with epinephrine and antihistamines, with complete resolution of his symptoms. His pea-specific serum IgE level was 8.75kU/L.

### Patient 2

A 3-year-old girl with a history of food allergy to egg and no other atopic diseases presented for evaluation of an allergic reaction to a ready macaroni and cheese meal containing peas and green beans. She passed an oral food challenge to green beans and was tolerating cheese and dairy well in her diet after the reaction. She failed an oral food challenge to green pea. Initially, she developed vomiting that was treated with cetirizine, followed by sneezing and right periorbital edema and erythema necessitating epinephrine. Her symptoms resolved over the course of an hour. Here pea-specific serum IgE level was 7.22kU/L.

### Patient 3

A 2-year-old boy with a medical history of allergic rhinitis with sensitization to tree, grass, ragweed, and dog and a history of fish and peanut food allergy presented to the clinic for evaluation of a reaction to green pea. He had perioral erythema 5 minutes after ingestion of green pea. The erythema resolved with the administration of Diphenhydramine HCL, and there were no other associated systemic symptoms. His pea-specific serum IgE level was 11kU/L.

### Patient 4

A 15-year-old boy who was previously healthy and had no atopic comorbidities other than a known history of cashew food allergy presented to the clinic for evaluation of new reactions occurring immediately after ingestion of different pea-containing food products. He tolerates peanuts without reactions. His first reaction was to Ripple milk (Ripple Foods, Emeryville, Calif), which is a pea-based milk that does not contain any peanuts or tree nuts, as specified on the label. The second reaction was to Beyond Meat chicken nuggets, which had the same ingredients listed as for the burger consumed by patient 1. The packaging also listed an advisory for pea food allergy, as it did on the burger ingested by patient 1. His last reaction was to a pea-based protein powder. The patient was unsure of the name of the product but was certain that it did not contain tree nuts, given his allergy. His reaction to all 3 ingestions was immediate hives located over his face and neck. He self-treated with Diphenhydramine HCL, with resolution of his symptoms. His pea-specific serum IgE level was 6.10 kU/L.

### Patient 5

A 2-year-old boy with a known allergy to peanut and no other atopic diseases presented for evaluation of a new allergic reaction associated with ingestion of a Chloe’s Oat Milk Mint Chip Ice Cream bar (Chloe's Fruit, New York, NY). Immediately after ingestion of the ice cream bar, he experienced perioral hives that were followed by throat clearing and lip swelling. The possible allergens listed on the ingredient list were pea protein, tapioca, flaxseed, sunflower lecithin, and chocolate chips. The product did not have an allergen declaration statement other than a warning that it contained nonallergenic highly refined coconut oil. He was eating and tolerating all ingredients except tapioca and pea, both of which he had never consumed before. He tested negative for tapioca serum IgE. His pea-specific serum IgE level was 31kU/L.

### Patient 6

A 7-year-old boy with a known medical history of eosinophilic esophagitis, allergic rhinitis with sensitization to tree, ragweed, and grass, and lentil food allergy presented to the clinic for follow-up. He had not been seen in the clinic for a while, and his mother mentioned that he had experienced a reaction to green peas at 18 months of age. The patient experienced facial angioedema and hives immediately after ingestion. His mother was unsure whether it was a “real” allergy, as she was not familiar with green peas being a food that can cause anaphylaxis. She had removed green peas from his diet out of caution but was not particular about reading labels. The boy's pea-specific serum IgE level was 73.4 kU/L.

## Discussion

The prevalence of pea allergies is largely unknown; according to a study of a German cohort, however, its sensitization rate was higher than that for soya beans, lupins, and peanuts.^9^ Also, peas are not recognized as a food that can trigger anaphylactic reactions by the public, as was the case with patient 6. The newfound popularity of pea protein in the food industry necessitates its careful consideration as a nonobvious allergen that can cause anaphylaxis, especially because it is regarded as an allergen alternative by the patient community. There are different nomenclatures for pea protein when listed as an ingredient. A Canadian case series examined different pea products causing allergic reactions and listed the following: pea protein hydrolysate, hydrolyzed pea protein, pea fiber, pea hull fiber,^10^ and vegetable protein.^3^

Peanuts and peas both belong to the legume family, which begs the question as to whether peanut and pea protein may exhibit cross-reactivity and whether there are other legumes with which pea protein may cross-react. Three of 6 of our patients showed concomitant peanut allergy and 1 also had a lentil allergy. Lentils also belong to the legume family. Our case series is too small to assume any association; however, further studies with a larger sample size are needed.

A few other publications have examined this question and reported the coexistence of legume and pea allergies. In 1 study by Wensing et al, 3 children with pea anaphylaxis had concurrent allergic reactions to peanuts, including oral symptoms, urticaria, and dyspnea.^9^ Those symptoms were explained by cross-reactive IgE initially formed against pea, and it was found that vicilin-like proteins in peanuts (*Arapis hypogaea 1*) and peas were identified as the cause of this cross-reactivity.^11^ In addition, some of these patients had reactions to other legumes, suggesting sensitization to pea vicilin-like protein-induced cross-reactivity among the legume family. Moreover, Sicherer postulated that allergy to 1 or more legumes could indicate a higher risk of several allergies.^11^

The importance of our case series lies in the fact that it highlights anaphylactic reactions to pea protein, which is typically considered safe and usually an alternative in those with other food allergies, as shown with patients 1 and 4. Our case series reiterates the need for more studies to define cross-reactivity between peas and peanuts, particularly as 3 of 6 of our patients had a concomitant peanut allergy and a fourth patient had a concomitant legume allergy. We were not able to exclude cross-contamination in the case of patient 5, as he has peanut allergy, which could very well have caused his reaction; however, given our experience with pea allergies and his serum IgE level, it was decided not to challenge to pea and opt for a safer option instead.

Our case series is too small to answer such questions, but we encourage allergists to be more mindful of pea protein as a relevant allergen and to evaluate pea allergy through skin testing or serum IgE testing if the patient presents with a concerning history. This is especially relevant in light of the popularity that pea protein is gaining. Further studies with a bigger sample size and more rigorous challenges are necessary to evaluate cross-reactivity within the legume family and, more importantly, whether the presence of a pea allergy should trigger evaluation for a peanut allergy and vice versa.
